# Identification and Validation of a Prognostic lncRNA Signature for Hepatocellular Carcinoma

**DOI:** 10.3389/fonc.2020.00780

**Published:** 2020-06-10

**Authors:** Wang Li, Qi-Feng Chen, Tao Huang, Peihong Wu, Lujun Shen, Zi-Lin Huang

**Affiliations:** ^1^Department of Medical Imaging and Interventional Radiology, Sun Yat-sen University Cancer Center, Guangzhou, China; ^2^State Key Laboratory of Oncology in South China, Guangzhou, China; ^3^Collaborative Innovation Center for Cancer Medicine, Guangzhou, China

**Keywords:** long non-coding RNAs, hepatocellular carcinoma, prognosis analysis, least absolute shrinkage and selection operator, TCGA

## Abstract

**Background:** An accumulating body of evidence suggests that long non-coding RNAs (lncRNAs) can serve as potential cancer prognostic factors. However, the utility of lncRNA combinations in estimating overall survival (OS) for hepatocellular carcinoma (HCC) remains to be elucidated. This study aimed to construct a powerful lncRNA signature related to the OS for HCC to enhance prognostic accuracy.

**Methods:** The expression patterns of lncRNAs and related clinical data of 371 HCC patients were obtained based on The Cancer Genome Atlas (TCGA). Differentially expressed lncRNAs (DElncRNAs) were acquired by comparing tumors with adjacent normal samples. lncRNAs displaying significant association with OS were screened through univariate Cox regression analysis and the least absolute shrinkage and selection operator (LASSO) algorithm. All cases were classified into the validation or training group at the ratio of 3:7 to validate the constructed lncRNA signature. Data from the Gene Expression Omnibus (GEO) were used for external validation. We conducted real-time polymerase chain reaction (PCR) and assays for Transwell invasion, migration, CCK-8, and colony formation to determine the biological roles of lncRNA. Gene set enrichment analysis (GSEA) of the lncRNA model risk score was also conducted.

**Results:** We identified 1292 DElncRNAs, among which 172 were significant in univariate Cox regression analysis. In the training group (*n* = 263), LASSO regression analysis confirmed 11 DElncRNAs including AC010547.1, AC010280.2, AC015712.7, GACAT3 (gastric cancer associated transcript 3), AC079466.1, AC089983.1, AC051618.1, AL121721.1, LINC01747, LINC01517, and AC008750.3. The prognostic risk score was calculated, and the constructed risk model showed significant correlation with HCC OS (log-rank *P*-value of 8.489e-9, hazard ratio of 3.648, 95% confidence interval: 2.238–5.945). The area under the curve (AUC) for this lncRNA model was up to 0.846. This risk model was confirmed in the validation group (*n* = 108), the entire cohort, and the external GEO dataset (*n* = 203). GACAT3 was highly expressed in HCC tissues and cell lines. Based on online databases, GACAT3 expression independently affects both OS and disease-free survival in HCC patients. Silencing GACAT3 *in vitro* significantly suppressed HCC cell proliferation, invasion, and migration. Moreover, pathways related to the lncRNA model risk score were confirmed by GSEA.

**Conclusion:** The lncRNA signature established in this study can be used to predict HCC prognosis, which could provide novel clinical evidence to guide targeted HCC treatment.

## Introduction

Hepatocellular carcinoma (HCC) is the most common form of liver cancer and has become a global health issue attracting wide attention (1). An increasing number of mutated genes have been implicated in HCC occurrence and development, including mammalian target of rapamycin, vascular endothelial growth factor (VEGF), and tumor protein (TP)53 (2, 3). However, HCC is a highly heterogeneous disease, which adds to the complexity in predicting prognosis. There is an urgent needed to identify novel biomarkers to diagnose HCC and precisely predict prognosis.

Compared with other cancer hallmarks, long non-coding RNAs (lncRNAs) show strong potential in making diagnosis and predicting prognosis thanks to several advantages. Firstly, lncRNA expression is highly variable among different disease stages, diseases, and tissues; as a result, it can better represent disease features (4). Secondly, lncRNAs can regulate gene expression at epigenetic, post-transcriptional, and transcriptional levels (5, 6); consequently, the functions and levels more closely correlate with tumor progression. Many studies have been performed to clarify the clinical value of lncRNAs within tumors including HCC (7). However, the existing lncRNA signatures for HCC prognosis require further optimization.

In this study, different lncRNA expression patterns were examined among appropriately selected HCC cases to identify candidate lncRNA biomarkers based on The Cancer Genome Atlas data (TCGA). The least absolute shrinkage and selection operator (LASSO) algorithm was used in determining key lncRNAs; thereafter, an HCC risk score system was also constructed and the lncRNA signature was validated. Finally, the roles of the target gene were validated *in vitro*. Our work yielded a signature based on lncRNA expression that can accurately predict HCC prognosis through integrated analysis of genomic data.

## Methods

### Patient Datasets and Processing

**Figure 1:**
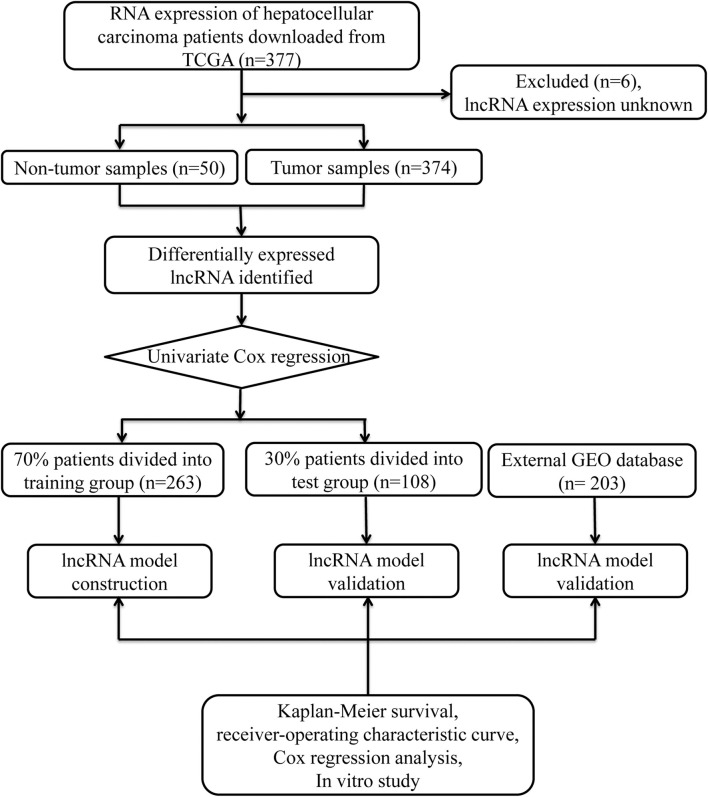
Overall study design.

Data from 377 HCC patients were downloaded from TCGA's database. The Data Transfer Tool of GDC Apps was utilized for downloading gene expression profiles and clinical information (https://tcga-data.nci.nih.gov/, accessed March 2019). Patients with unknown lncRNA expression were excluded (*n* = 6), leaving 371 HCC cases in the final cohort for analysis (Table 1). Figure 1 displays the analysis flow chart. A total of 371 HCC patients were randomly divided into the validation or training group at the ratio of 3:7 for integrated analysis using the “caret” package (Supplementary Tables 1, 2). Both training and test cohorts were required to meet the following criteria: (1) samples were randomly assigned to training and testing cohorts; (2) the clinical features of subjects in these groups were similar. All data were publicly available and open-access, so it was unnecessary to obtain Ethics Committee approval. Data were processed in accordance with the NIH TCGA human subject protection (http://cancergenome.nih.gov/publications/publicationguidelines) and related data access policies.

**Table 1:** Baseline data of all HCC patients.

**Characteristic**		***n***	**Proportion (%)**
Total		371	100
Median follow-up (days)	557 (1–3,675)	371	100
Age	59.5 ± 13.0	371	100
Sex	Male	251	67.7
	Female	120	32.3
Race	White	230	62.0
	Others	141	38.0
Tumor grade	I	54	14.6
	II	178	48.0
	III	122	32.9
	IV	12	3.2
	Unknown	5	1.3
Stage	I	174	46.9
	II	86	23.2
	III	84	22.6
	IV	5	1.3
	Unknown	22	5.9
T stage	I	183	49.3
	II	94	25.3
	III	80	21.6
	IV	12	3.2
	Unknown	2	0.5
N stage	Without metastasis	254	68.5
	With metastasis	4	1.1
	Unknown	113	30.5
M stage	Without metastasis	268	72.2
	With metastasis	4	1.1
	Unknown	99	26.7

The lncRNA expression among HCC cases was derived from the Illumina HiSeq RNASeq platform (Illumina, San Diego, CA, USA), which was standardized using TCGA. To examine differential RNA expression, the R software “edgeR” package was utilized for identifying differentially expressed lncRNAs (DElncRNAs), and the thresholds were set as |log_2_ foldchange (FC) | >2.0, with an adjusted *P* < 0.05.

### lncRNA Signature Construction

The relationship of lncRNA expression with overall survival (OS) was calculated with univariate Cox modeling. lncRNA expression differences were considered statistically significance at *P* < 0.05. For the training group, the screened lncRNAs were further selected and validated through LASSO regression using the R project “glmnet” package. Finally, the lncRNA-based prognosis risk score was established on the basis of linearly combining the formula below with the expression level multiplied regression model (β). Risk score = βlncRNA1 × lncRNA1 expression + βlncRNA2 × lncRNA2 expression + · ···· +βlncRNAn × lncRNAn expression. We also compared the model lncRNA transcriptomic profiles from HCC and normal tissue samples using TCGA and The Genotype-Tissue Expression (GTEx) data.

### Confirmation of the lncRNA Signature

Cases together with their survival information were distributed according to the risk score. Cases were also classified according to the median risk score threshold as high or low risk, and Kaplan–Meier survival curves were plotted for both groups. Thereafter, the univariate Cox proportional hazards regression modeling was employed. The time-dependent receiver operating characteristic (ROC) curves were then used to evaluate the prognostic value, which was achieved through comparing the specificity and sensitivity in predicting survival on the basis of risk score. In addition, multivariate Cox regression was conducted to verify the relationship of lncRNA risk score prediction with other clinical parameters. The predictive accuracy of the lncRNA model was then verified in the validation group (*n* = 108). The GSE14520-GPL3921 dataset from the GEO database was used as an independent validation cohort (*n* = 203). Each test was two-sided, and *P* < 0.05 was deemed statistically significant. R software (version 3.6.0; R Foundation) was adopted for all analyses.

### Cell Culture and Tissue Specimens

Three human HCC cell lines (MHCC-97H [97H], HepG2 [G2], and MHCC-LM3 [LM3]) were purchased from the Cell Bank of the Type Culture Collection of the Chinese Academy of Sciences, Shanghai Institute of Biochemistry and Cell Biology. The cell lines were all cultured in Dulbecco's minimum essential media (DMEM) plus 10% fetal bovine serum (FBS; Invitrogen, Carlsbad, CA, USA). All cell lines were grown without antibiotics in a humidified atmosphere of 5% CO_2_ and 99% relative humidity at 37°C. Three different HCC cell lines and 26 fresh HCC tumor samples paired with their paratumor tissues were subjected to quantitative real-time polymerase chain reaction (qRT-PCR). This study was approved by our medical institution's Ethics Committee.

### RNA Isolation and qRT-PCR Analysis

Total cellular RNA was extracted using TRIzol reagent (Invitrogen). First-strand cDNA was synthesized using random primers. The relative RNA expression levels were determined by qRT-PCR in triplicate on a Bio-Rad CFX96 system (Bio-Rad, Hercules, CA, USA) using the SYBR Green method. The primers were as follows: GACAT3 forward ACAGGCTTTGGTTTCAGGACA, GAPDH forward CCCATCACCATCTTCCAGGAG, GAPDH reverse GTTGTCATGGATGACCTTGGC, and GACAT3 reverse CTGTCCTATGCGCTGGTGAT. Quantifications were normalized by using glyceraldehyde 3-phosphate dehydrogenase RNA as an internal reference and calculated using the comparative Ct method.

### Transwell Migration and Wound Healing Assays

Migration assays were performed in a 24-well Millicell chamber. Briefly, 97H (5 × 10^5^ cells per well), G2 (2.5 × 10^4^ cells per well), and LM3 (2.5 × 10^4^ cells per well) cells in 200 μl of serum-free medium were added to coated filters. Then, 700 μl of medium containing 20% FBS was placed in the lower chamber. After different times in an incubator at 37°C, the cells that migrated through the filter were fixed with methanol, stained with 0.5% crystal violet, and counted in three random fields. The invasion assay were conducted using 8-μm pore inserts coated with 30 μg of matrigel (BD Biosciences). 97H (1 × 10^5^ per well), G2 (5 × 10^4^ per well), and LM3 (5 × 10^4^ per well) were added to the coated filters. Additionally, 97H, G2, and LM3 cells were cultured in 6-well-plates and scraped with a 200-μl pipette tip. The cells were cultured in DMEM without FBS. Cell migration was photographed using an inverted microscope (OLYMPUS IX73, Olympus, Tokyo, Japan) at 0 and 24 h after injury.

### Cell Viability and Colony Formation Assays

Briefly, 97H (1 × 10^3^ cells per well), G2 (1.5 × 10^3^ cells per well), and LM3 (1 × 10^3^ cells per well) cells were seeded in 96-well-plates. After different incubation times, cell viability was measured with the Cell Counting Kit-8 (CCK-8, Dojindo, Kumamoto, Japan). Regarding colony formation experiment, 1,000 cells were seeded in cell culture plates and allowed to grow until visible colonies formed. Cell colonies were fixed with methanol, stained with crystal violet, and counted.

### Functional Analysis

Underlying mechanisms were investigated within “Molecular Signatures Database” of c2.cp.kegg.v6.2.symbols through gene set enrichment analysis GSEA (8) with a Java program (http://software.broadinstitute.org/gsea/index.jsp). The random sample permutation number was set as 1,000, and the significance threshold was *P* < 0.05.

## Results

### DElncRNAs Identification

**Figure 2:**
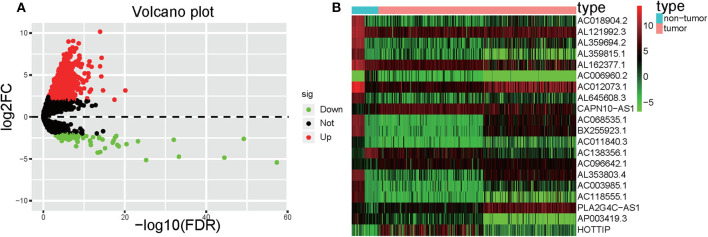
Volcano plot and heatmap. **(A)** Volcano plot depicting the DElncRNAs; the *X*-axis represents the log-transformed values of false discovery rates, and the *Y*-axis indicates the average differences in lncRNA expression. Red and green dots indicate the up- and downregulated lncRNAs in tumor, and black dots indicate DElncRNA with nonsignificant differences. **(B)** Heatmaps demonstrate the DElncRNAs; the *X*-axis shows the sample category, and the *Y*-axis represents the DElncRNAs. Green and red indicate down- and up-regulation, respectively.

Significant DElncRNAs were identified among tumor samples compared with non-tumor samples. A total of 1292 DElncRNAs (80 downregulated and 1,212 upregulated) were identified using the R project “edgeR” package. These data were used to build the volcano plot of DElncRNAs (Figure 2A) and the heat map of the top 20 genes (Figure 2B).

### Construction of the lncRNA Signature

**Figure 3:**
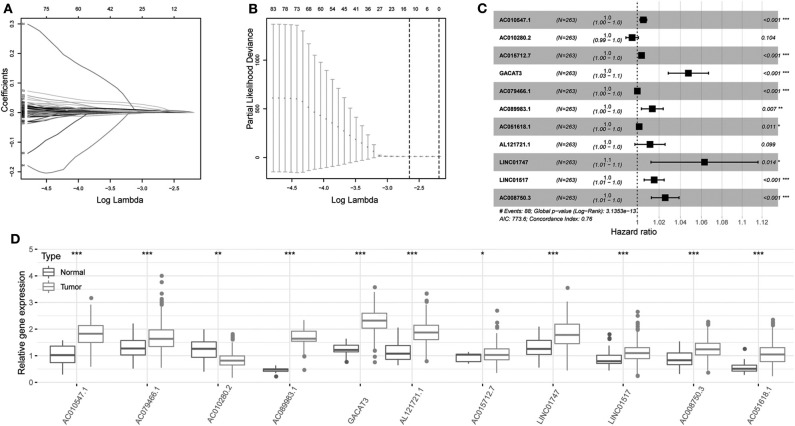
Regression coefficient diagram based on LASSO regression. **(A)** LASSO coefficient profiles for some significant lncRNAs in univariate Cox regression analysis. Coefficient profiles decrease with larger lambda values. **(B)** Cross-validation for selecting the tuning parameters for the LASSO model. The vertical lines are plotted based on the optimal data according to the minimum criteria and 1-standard error criterion. The left vertical line represents the 11 lncRNAs finally identified. **(C)** Forest plots showing the relationships of various lncRNA subsets with OS in training cohort. The unadjusted HRs are presented with 95% CIs. **(D)** Differential gene expression of model lncRNA in TCGA and GTEx database. ***P < 0.001, **P < 0.01, and *P < 0.05.

Univariate Cox regression was carried out between DElncRNAs and OS, and the results showed that a total of 172 DElncRNAs were significantly related to OS (*P* < 0.05. Next, LASSO regression was employed for verifying further variables in the training cohort (Figures 3A,B). Eleven lncRNAs were produced in this process, including AC010547.1, AC010280.2, AC015712.7, GACAT3 (gastric cancer associated transcript 3), AC079466.1, AC089983.1, AC051618.1, AL121721.1, LINC01747, LINC01517, and AC008750.3. Figure 3C shows the forest plot of the relationships of every lncRNA with OS. Then, the following prognostic risk score was calculated: (1.0055 × AC010547.1 expression) + (0.9953 × AC010280.2 expression) + (1.0039 × AC015712.7 expression) + (1.0475 × GACAT3 expression) + (1.0001 × AC079466.1 expression) + (1.0137 × AC089983.1 expression) + (1.0017 × AC051618.1 expression) + (1.0116 × AL121721.1 expression) + (1.0630 × LINC01747 expression) + (1.0154 × LINC01517 expression) + (1.0257 × AC008750.3 expression). Comparison of transcriptome profiles from TCGA and GTEx found that the expression of most lncRNAs was markedly upregulated in HCCs, which is presented in Figure 3D.

### Confirmation of the lncRNA Signature

**Figure 4:**
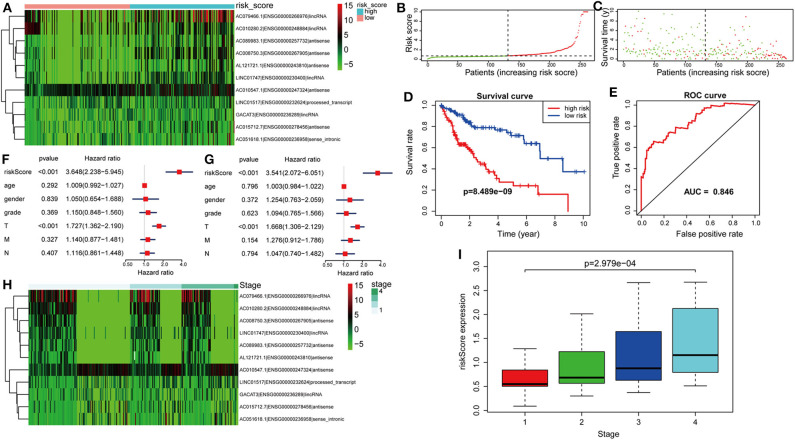
Verification of the lncRNA signature for predicting HCC prognosis in the training group. **(A)** LncRNA expression in the high- and low-risk groups. **(B)** Distribution of lncRNA risk score. **(C)** Survival status together with OS. **(D)** Kaplan–Meier curve showing OS in the low- and high-risk groups classified based on the median risk score. **(E)** The ROC curve of survival discriminated by the lncRNA signature. **(F)** Univariate Cox regression analyses of OS. **(G)** Multivariate Cox regression analyses of OS. **(H)** LncRNA expression grouped by pathological stage. **(I)** Risk score significantly increased with more advanced stage.

The risk score was computed for every case, and all cases were classified as low or high risk based on the median threshold. The distributions of 11 lncRNA expression levels together with groups are shown in Figure 4A. Figures 4B,C display the distributions of risk score and survival time in the training group, respectively. Figure 4D presents the Kaplan–Meier curves for the low- and high-risk groups. Cases with high risk scores had shorter OS compared with low-risk cases (*P* = 8.489e−9). Time-dependent ROC curves were utilized in assessing the performance of lncRNA biomarkers in prognosis prediction. In addition, the area under the curve (AUC) for the as-constructed lncRNA biomarkers-based prognostic model was 0.846 (Figure 4E). Besides, the hazard ratio (HR) for risk score upon univariate Cox proportional hazards regression was 3.648 (95% confidence interval [CI]: 2.238–5.945; Figure 4F). Consistent results were obtained through multivariate Cox proportional hazards regression (HR = 3.541, 95% CI: 2.072–6.051) adjusted for the clinical covariate (Figure 4G). Figure 4H shows 11 lncRNA expressions grouped by pathological stages. The risk score increased significantly with advanced pathological stage (*P* = 2.979e−4, Figure 4I).

### Validation of the lncRNA Model

**Figure 5:**
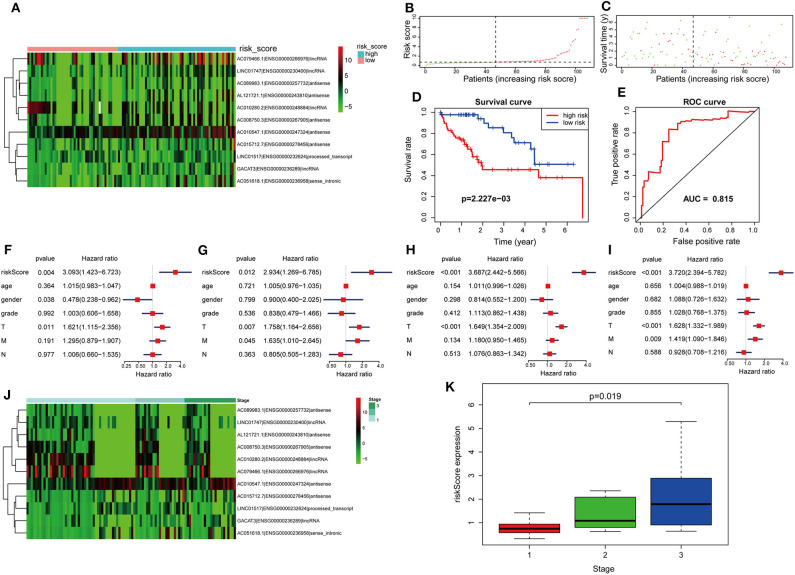
Further verification of the lncRNA signature for HCC prognosis in the validation group and the entire cohort. **(A**–**E)** are validation group results that are consistent with the training cohort results (Figure 4). Cox regression results. **(F)** Univariate results in the validation group. **(G)** Multivariate results in the validation group. **(H)** Univariate results for the entire cohort. **(I)** Multivariate results for the entire cohort. **(J)** LncRNA expression grouped by pathological stage in the validation group. **(K)** Risk score significantly increased for advanced stage cases in the validation group.

The formula was further used in the entire cohort and validation cohort to verify the similar prognostic significance of the as-constructed lncRNA model among distinct populations. Figures 5A–C show the distributions of lncRNA expressions, risk score, and survival time in the validation group, respectively. Figure 5D presents the Kaplan–Meier curves for the low- and high-risk groups. The OS for patient benefited from low-risk score in the validation group (*P* = 2.227e−3). The AUC for the validation group was 0.815 (Figure 5E). In line with results obtained from training cohort, the lncRNA model was an independent prognostic factor in univariable and multivariable analyses for the validation cohort and entire cohort (Figures 5F–I). Figure 5J shows 11 lncRNA expressions grouped by pathological stages; the risk score was significantly higher for advanced pathological stage (*p* = 0.019, Figure 5K) in the validation cohort.

**Figure 6:**
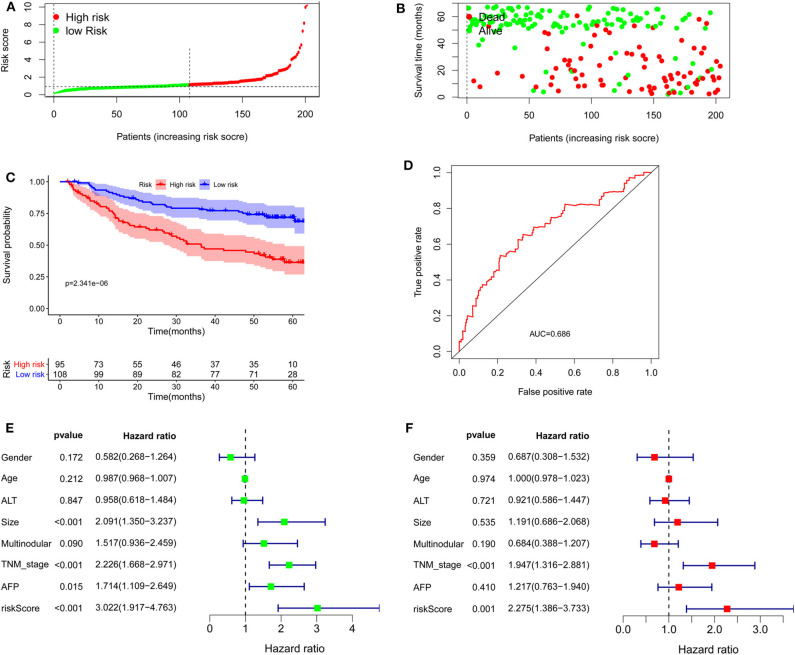
Validation of the lncRNA signature in the Gene Expression Omnibus cohort. **(A)** Distribution of lncRNA risk score. **(B)** Survival status together with OS. **(C)** Kaplan–Meier curves of overall survival. **(D)** Time-dependent receiver operating characteristic curves. **(E)** Univariate and **(F)** multivariate Cox regression analysis further confirmed the signature as an independent factor.

To confirm the external validity, the model was applied in the external GEO data. Figures 6A,B show the distributions of risk score, and survival time in the GEO validation group. The high-risk group had a significantly shorter survival than the low-risk group in the GEO cohorts (*P* = 2.341e−6; Figure 6C). ROC curve analysis showed that risk signature prognosis prediction could attain an AUC value of 0.686 (Figure 6D). Univariate (*P* < 0.001) and multivariate (*P* < 0.001) Cox regression analysis confirmed the signature was an independent prognostic factor (Figures 6E,F).

### GACAT3 Is Highly Expressed in HCC Tissues and Correlates With Poor Prognosis

**Figure 7:**
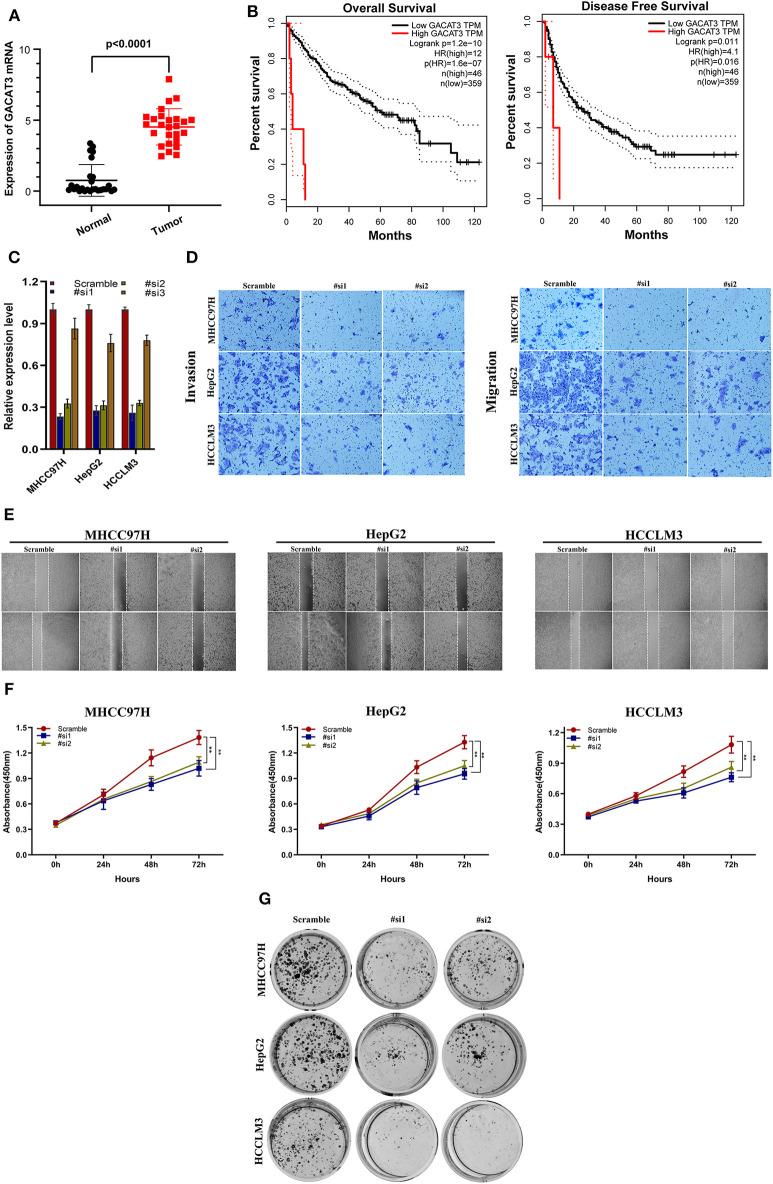
The clinical significance of GACAT3 in HCC and *in vitro* study. **(A)** GACAT3 are overexpressed in HCC tissues, and higher GACAT3 level predicts poor prognosis **(B)**. **(C)** Transfection efficiency was verified after transfection of GACAT3 or negative control siRNA. **(D)** Transwell assays were used to detect HCC invasion and migration. Representative experiments are shown. **(E)** Images were recorded 0 and 24 h after scratching the cell surface; representative images are shown; **(F)** HCC cell viability was evaluated with CCK-8 assays at 0, 24, 48, and 72 h post-transfection. **P < 0.001. **(G)** The number of HCC cell colonies was reduced after GACAT3 knockdown.

GACAT3 expression was the most upregulated of the prognostic lncRNAs. Therefore, the role of GACAT3 with regards to HCC was further assessed. Using qRT-PCR, we evaluated GACAT3 expression levels in 26 HCC tissues and paired adjacent normal tissues. GACAT3 mRNA expression was higher in HCC tissue compared to adjacent normal liver (*P* < 0.0001, Figure 7A). To assess the potential prognostic ability of GACAT3 in HCC patients, gene expression profiling interaction analysis (GEPIA) (http://gepia.cancer-pku.cn/) was used for survival analysis. As shown in Figures 7B, lower GACAT3 was associated with longer OS (*P* = 1.2e−10) and better disease-free survival rates (*P* = 0.011).

### Downregulation of GACAT3 Inhibits HCC Cell Migration and Proliferation

We first evaluated the transfection efficiency of the cells by qRT-PCR and found that the relative expression level of GACAT3 was significantly lower after siRNA 1 and 2 transfection (Figure 7C). To further confirm the role of GACAT3 in invasion and migration, Transwell assays were performed. Our results showed that the invasion and migration rates of 97H, G2, and LM3 cells transfected with siRNA were significantly lower than that of the control-transfected cells (Figure 7D). Wound healing assays revealed that silencing GACAT3 significantly repressed wound healing in all three cell lines (Figure 7E). We performed CCK-8 assays to detect the effect of GACAT3 knockdown on cell proliferation. After GACAT3 silencing, 97H, G2, and LM3 cell proliferation significantly decreased compared to control cells (Figure 7F, *P* < 0.001). Colony formation assay also indicated that GACAT3 silencing significantly suppressed the growth of all three cell cells (Figure 7G). These data suggest that GACAT3 knockdown repressed the proliferative, migratory, and invasive abilities of 97H, G2, and LM3 cells.

### Functional Analysis of Prognostic lncRNA Model

**Figure 8:**
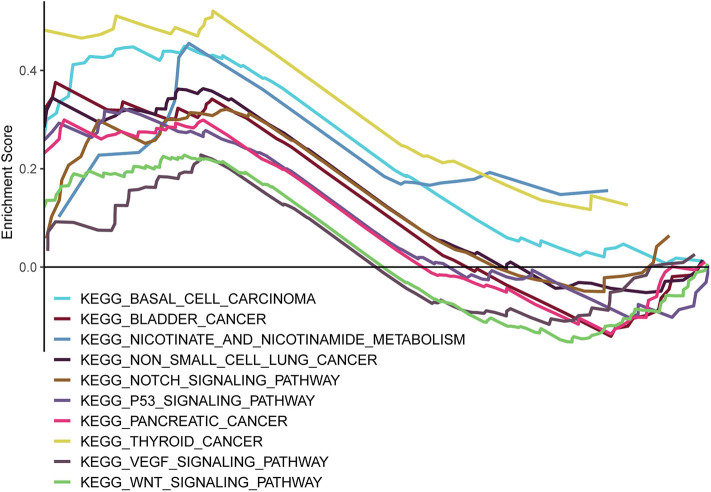
GSEA delineation of the biological pathways related to the risk score values of the lncRNA model using the gene set “c2.cp.kegg.v6.2.symbols”.

GSEA was carried out to examine the biological effects of the as-constructed lncRNA model, and our results suggested that the high score of lncRNA model showed significant enrichment in pathways including, bladder cancer, basal cell carcinoma, non-small cell lung cancer, nicotinamide and nicotinate metabolism, the notch signal transduction pathway, the p53 signal transduction pathway, thyroid cancer, pancreatic cancer, the VEGF signal transduction pathway, and the Wnt signal transduction pathway (Figure 8).

## Discussion

A small number of lncRNA-based prognostic models have been specifically developed for HCC. To our knowledge, little is known concerning the lncRNA signature for HCC patients. Gu and colleagues constructed a six-lncRNA signature to predict HCC recurrence-free survival, while Wu et al. performed analysis in a specified resectable HCC population (9, 10). Different from previous publications, this study was performed in HCC patients to predict survival using an lncRNA signature. In this study, OS-associated DElncRNAs were comprehensively screened by applying the biostatistics method and univariate Cox analysis. Then, LASSO regression was applied in lncRNA data from TCGA, and 11 lncRNAs were filtered out. Significant lncRNAs were utilized in constructing the prognostic model. Then, Kaplan–Meier analysis, Cox regression analysis, and the time-dependent ROC curves were employed to confirm the prognostic significance of the lncRNA signature, which was recognized to be an independent factor to predict HCC prognosis. Further validation was carried out in both the internal and external validation cohorts.

GACAT3 was closely associated with gastric cancer in previous studies. Lin et al. (11) found that knockdown of GACAT3 significantly decreased gastric cancer cell proliferation. Feng et al. (12) reported that higher GACAT3 levels were significantly associated with shorter OS in gastric cancer patients, and knockdown of GACAT3 significantly inhibited gastric cell functions *in vitro*. Overexpression of GACAT3 in lung cancer cells promoted cell proliferation and migration (13), and it enhanced their sensitivity to radiotherapy. GACAT3 was recently demonstrated to promote progression of colorectal cancer (14), breast cancer (15), and glioma (16, 17). However, the role of GACAT3 in HCC remains unclear. The present study shows that GACAT3 is upregulated in HCC tissues, could serve as a poor predictor of HCC patients, and promotes progression in cell lines. However, the underlying mechanism needs to be explored in future studies.

Compared with existing articles that examined the lncRNA prognostic effects on HCC, some strengths of this study should be noted. Firstly, all HCC patients in TCGA were enrolled for analysis, and the total sample size was considerable. Secondly, with regard to methodology, LASSO penalized regression was applied to increase the accuracy of the bioinformatic analysis. Different from conventional stepwise regression employed in prior articles, the LASSO algorithm is able to simultaneously analyze each independent variable, and it tends to select the variables of the highest significance (18). Notably, the less significant variable has a correlation coefficient of 0 following introduction of a penalty in accordance with the regularized path (19). Consequently, this approach achieves much higher accuracy than multivariate Cox model stepwise regression; particularly in the case of processing large datasets, such as genomics data (20). Thirdly, the lncRNA signature was produced in a training group, and the model was validated internally and externally, underscoring the reliability of the results.

Pathway enrichment indicated the above lncRNAs potentially affected HCC occurrence and progression via 10 pathways, and their biological effects on HCC had been reported in published articles. Some of them were canonical and important pathways related to HCC initiation and development. The biological effects of those determined lncRNAs on HCC have not been investigated or reported. However, our pathway enrichment results suggested the potential influence of these lncRNAs on HCC occurrence and progression via the notch (21, 22), p53 (23, 24), VEGF (25, 26), or Wnt signal transduction pathways (27–31). These findings offer new evidence to support that lncRNAs whose biological functions have not been reported in published articles could potentially serve as HCC prognostic predictors. Nonetheless, these results should be validated in future studies, and the molecular characteristics should also be investigated.

There are several acknowledged limitations in this study. Firstly, *in vivo* or further *in vitro* experimental study was not carried out to validate the prognostic performance of our proposed lncRNA signature for HCCs; instead, it was deduced based on online datasets through bioinformatic approaches. Secondly, Russi et al. (32) found that global gene expression profile of normal tissue adjacent to the tumor are characterized by a peculiar biological behavior different from both healthy and tumor tissues. Therefore, our results require further validation.

In conclusion, we identified a novel lncRNA signature that could be an independent biomarker for predicting HCC prognosis through comprehensive bioinformatic analysis in combination with clinical information and genetic profiles of a carefully screened cohort. Nevertheless, our results should be validated in future studies that examine HCC progression mechanisms as well as the effects of these 11 lncRNAs.

## Data Availability Statement

The datasets generated for this study can be found in the https://portal.gdc.cancer.gov.

## Ethics Statement

This research project was approved by the Ethics Committee of Sun Yat-sen University Cancer Center.

## Author Contributions

WL, Q-FC, and Z-LH conceived of and designed the study. WL, Q-FC, PW, and TH performed the literature search, generated the figures and tables, and wrote the manuscript. Q-FC, LS, and Z-LH collected and analyzed the data, and critically reviewed the manuscript. Q-FC, WL, and Z-LH supervised the study and reviewed the manuscript.

## Conflict of Interest

The authors declare that the research was conducted in the absence of any commercial or financial relationships that could be construed as a potential conflict of interest.

## Supplementary Material

Supplementary Table 1 lncRNA signature and clinical data in the training group.

Supplementary Table 2 lncRNA signature and clinical data in the validation group.
